# A survey of antibiotic administration practices involving patients with sepsis in UK critical care units

**DOI:** 10.1007/s11096-019-00938-9

**Published:** 2019-11-14

**Authors:** Gregory J. Barton, Charles W. Morecroft, Neil C. Henney

**Affiliations:** Pharmacy & Biomolecular Sciences, Liverpool John Moores University, Liverpool, UK

**Keywords:** Antibiotic administration, Clinical outcome, Critical care, Critical care pharmacist, Sepsis, Therapeutic drug monitoring, United Kingdom

## Abstract

*Background* Alternative administration methods are emerging as a key area of research to improve clinical efficacy of antibiotics and address concerns regarding multi-drug resistance. Extended intermittent infusions or continuous infusions of antibiotics exhibiting time-dependent kill characteristics may be favourable in critically ill septic patients, but more evidence is needed to determine best practice. *Objective* To find out whether any common practice exists for intravenous antibiotic administration in critical care units across UK NHS Trusts, and identify factors influencing the adoption of extended or continuous infusions. *Setting* UK hospitals. *Method* UK critical care pharmacists were invited to participate in a survey on behalf of all 240 critical care units via a UK Clinical Pharmacy Association message board. The survey focused on administration practices for 22 antibacterial agents. *Main outcome measure* Antibiotic administration method. *Results* Responses were received covering 64 units, a response rate of 26.2%. Common, but not uniform administration methods were apparent for 17/22 antibiotics. Four antibiotics (piperacillin/tazobactam, doripenem, meropenem and vancomycin) were more likely to be administered as continuous or extended-intermittent infusions. Choice of administration method was especially influenced by altered pk/pd properties in sepsis or severe burns patients, or by the presence of organisms requiring high minimal inhibitory concentrations. *Conclusion* Unlicensed alternative practices of antibiotic administration are widespread but only weak evidence exists of any patient benefit, such as reduced length of stay in critical care, and none showing improvement in mortality. Further research is needed to determine whether extended infusion methods offer clinically meaningful advantages over shorter licenced administration methods in patients in critical care units.

## Impacts on practice

- Administration method of antibiotics in practice often does not strictly follow guidance provided by the summary of product characteristics for each antibiotic, meaning practitioners take responsibility for deviating from the licensed method.
- The apparent lack of “standard” practice for the administration of antibiotics demonstrates that there is a need for evidence synthesis and evidence-based guidance to try to improve a number of important clinical and efficiency outcomes in critical care.

## Introduction

Antibiotic selection in UK hospitals is largely guided by local policy and guidelines that have been developed for common organisms or infection sites and with resistance and susceptibility patterns in mind. Antibiotic policies generally state the antibiotic to be prescribed, along with the dose, frequency and route of administration but rarely specify a method of administration, e.g. how quickly a bolus should be given or the time over which an infusion should run. Given the escalating global concern regarding multi-drug resistant organisms, alternative methods of administration and dosing strategies are becoming a key area of research interest to improve clinical efficacy of available antibiotics. A growing body of published evidence [1, 2] shows that in the critically ill septic patient extended intermittent infusions (EIIs) or continuous infusions (CIs) of antibiotics exhibiting time-dependent kill characteristics may be favoured because of their pharmacokinetic/pharmacodynamic (pk/pd) properties, but more evidence is needed to determine best practice.

In the intensive care setting infection and related sepsis is the leading cause of death with a mortality rate of up to 60% [3]. Although clinical guidance exists for some aspects of antibacterial therapy such as timing (e.g. in “sepsis 6”) and which classes of antibacterial should be used [4, 5], at the time of the survey no guidance existed specifying methods of intravenous antibacterial administration, although this has since changed [5].

Two recent clinical trials [1, 6] have shown an increase in clinical cure rates with continuous infusions, whereas another trial [7] failed to show improvement, but whether alternative administration methods change the risk of mortality in critically ill patients remains unknown. The ADMIN-ICU survey [8] of antibiotic dosing and monitoring in ICUs showed very diverse practices in administration of selected antibacterial agents from responding sites across 53 countries, and concluded that further research to develop best practice guidelines is required.

## Aim of the study

To find out whether any ‘usual’ (common) practice exists for intravenous antibiotic administration in critical care units across UK NHS Trusts, and identify factors influencing the adoption of extended or continuous infusions of antibiotics.

## Ethics approval

Ethics approval for this study was obtained from the Liverpool John Moores University Research Ethics Committee (13/SPS/044). Research was conducted in accordance with the Declaration of Helsinki and UK/EU and institutional standards. Informed consent was obtained from all individual participants included in the study.

## Method

### Study design

A survey was designed to collect data on: 1. pertinent demographic information of the responding pharmacists and CCUs (specialty, seniority, grade, experience and CCU microbiologist cover); 2. the usual method of intravenous administration of each of 22 antimicrobial agents; 3. any alternative methods being used to administer intravenous antibiotics; 4. the driving forces for selection of certain intravenous administration practices.

The survey was distributed using the ‘Bristol Online Surveys’ tool and responses were collected using a variety of methods, including drop-down menu options and radio buttons with some free-text options, which were later grouped by themes. Choice of the topics and questions for inclusion/exclusion in the survey was guided by personal experience of the subject matter and the published literature to identify themes and relevant antibiotics.

Participant anonymity was maintained as far as possible during data collection, and details from individual NHS Trusts are not shown as the aim of the study was to look at UK trends not individual Trust practice. The survey was piloted with a small number of critical care pharmacists via a secure message board, and amendments made according to their reponses and suggestions.

Participants (UK hospital critical care pharmacists) were invited to complete the survey via the UKCPA CCG message board, during an 8-week period between October and December 2013. Two follow-up reminder messages were posted 2 and 6 weeks after the initial invitation. There were 244 critical care units (CCUs) in the UK at the time of the survey and 600 pharmacists registered with the UKCPA CCG message board. A list of CCUs was obtained from the Intensive Care National Audit & Research Centre (ICNARC) and the Scottish Intensive Care Societies Audit Group (SICSAG) and all units listed with a pharmacist member of the UKCPA CCG were invited to participate. Duplicate responses from individual Trusts were removed by using only the response given by the most senior pharmacist, on the basis that they would potentially be more knowledgeable about the practices than junior pharmacists.

For the purposes of this study, “usual practice” was defined as existing when an antibiotic was administered by the same method on greater than 50% of responding CCUs. Data values were exported into Microsoft^®^ Excel^®^ (for Mac^®^ 2011, v. 14.6.2) and statistical analysis was performed using IBM^®^ SPSS^®^ Statistics 21.0. Demographic data were prepared as distribution frequencies and percentages, as were data comparing antibiotics, the method of administration, the stated factors influencing practice and the rate of TDM. The Chi squared test was used to assess differences between groups. Statistical significance level was set at α = 0.05.

## Results

The questionnaire asked for the pharmacist responding to do so for all the CCUs that they worked on, as some cover multiple CCUs within the same NHS Trust. In total, 54 pharmacists responded on behalf of 64 CCUs, a response rate of 26.2%.

90.6% (58/64) of responding CCUs were from England, 5 from Scotland and 1 from Northern Ireland, and at least one unit responded from the majority of Critical Care Networks (CCNs)/regions in the UK as defined by ICNARC/SICSAG. Table 1 shows a summary of the characteristics of responding pharmacists and their associated CCUs.

**Table 1:** Characteristics of responding critical care units

		n	(%)
Country	England	58	(90.6)
	Wales	0	(0)
	Scotland	5	(7.8)
	Northern Ireland	1	(1.6)
Specialty of CCU	Surgical	1	(1.6)
	General/mixed	60	(93.7)
	Cardiothoracic	1	(1.6)
	Other*	2	(3.1)
Size of CCU	< 10 beds	14	(21.9)
	10–20 beds	31	(48.4)
	> 20 beds	12	(18.8)
	Not known	7	(10.9)
CCU pharmacist grade (*agenda for change* band)	7	6	(9.4)
	8a	31	(48.4)
	8b–9	27	(42.2)
Pharmacist years experience in CCU	< 1	2	(3.1)
	1–5	11	(17.2)
	6–10	19	(29.7)
	> 10	32	(50.0)
CCU pharmacist cover	Never	0	(0)
	Rarely/ad hoc	1	(1.6)
	Weekdays	52	(81.3)
	Weekdays and Saturdays	0	(0)
	Everyday	11	(17.2)
Pharmacist attending consultant-led CCU ward round	Yes	47	(73.4)
	No	17	(26.6)
Medical microbiologist CCU ward rounds	None	0	(0)
	Weekly	13	(20.3)
	Weekdays	41	(64.1)
	Everyday	10	(15.6)

*Burns/trauma × 1, complex respiratory × 1

### Administration and monitoring practices

The usual administration method of 22 antibacterial agents was ascertained for each CCU (Table 2). Of these, only 4 antibiotics were given by a single administration method across all responding CCUs (ticarcillin/clavulanic acid, imipenem/cilastatin, tigecycline, and ciprofloxacin). However, frequency analysis showed that 17/22 antibiotics had a single preferred method of administration used in greater than 50% of the responding CCUs. Four antibiotics were administered on at least 20% of CCUs by EII or CI: piperacillin/tazobactam, doripenem, meropenem and vancomycin. Doripenem was reportedly only used in 3 of the responding CCUs, and ampicillin in only 2, so were not included in further analyses. Piperacillin/tazobactam and meropenem were administered as EIIs in 22.2% and 20.3% respectively of responding CCUs, and vancomycin by CI in 48.4% of CCUs. Therapeutic drug monitoring, which serves to influence dosing and administration methods, was reportedly done for only glycopeptide and aminoglycoside agents (100% of CCUs) but no other agents.

**Table 2:** Reported antibacterial administration method

	Usual method of administration per CCU, *n* (%)						
	B	S/B	S/SPC	EII	CI	N/A	No. of CCUs
Benzylpenicillin	30 (48.4)	6 (9.7)	22 (35.5)		4 (6.4)	2	62
Flucloxacillin	30 46.9)	8 (12.5)	22 (34.4)		4 (6.2)		64
Amoxicillin	38 (65.5)	8 (13.8)	12 (20.7)			6	58
Ampicillin	1 (50)		1 (50)			62	2
Co-amoxiclav	40 (67.8)		13 (22)	6 (10.2)		5	59
Piperacillin/tazobactam	17 (27)		32 (50.8)	14 (22.2)		1	63
Ticarcillin/clavulanic acid			10 (100)			54	10
Cefotaxime	20 (60.6)	2 (6.1)	11 (33.3)			31	33
Ceftazidime	20 (40.8)	1 (2)	20 (40.8)	8 (16.4)		15	49
Ceftriaxone	22 (36.7)	1 (1.7)	33 (55)	4 (6.6)		4	60
Cefuroxime	24 (61.5)		11 (28.2)	4 (10.3)		25	39
Doripenem			2 (66.7)	1 (33.3)		61	3
Ertapenem	5 (13.2)		33 (86.8)			26	38
Imipenem/cilastatin			5 (100)			59	5
Meropenem	29 (45.3)	4 (6.3)	18 (28.1)	13 (20.3)			64
Tigecycline			38 (100)			26	38
Clarithromycin			63 (98.4)	1 (1.6)			64
Clindamycin			62 (96.8)	2 (3.2)			64
Vancomycin			32 (50.8)		31 (49.2)	1	63
Teicoplanin	34 (57.6)		25 (42.4)			5	59
Linezolid			60 (96.7)	2 (1.7)		2	62
Ciprofloxacin			64 (100)				64

B, bolus injection, i.e. over 5 min or less; S/B, short infusion as per the SPC or bolus injection (dependent on dose); S/SPC, short infusion as per the SPC; EII, extended intermittent infusion, i.e. the drug is infused over a period that is longer than that suggested in the SPC; CI, Continuous infusion, i.e. the drug is infused continuously for the duration of the treatment course; N/A, agent not used

### Factors affecting choice of administration method including alternative dosing methods

A number of factors affected the adoption of EII or CI for each antibiotic. Higher pharmacist grade (*p *= 0.028), greater pharmacist cover (*p *< 0.001) and greater microbiologist input (*p *= 0.031) were associated with the adoption of EIIs as the usual method of administration of piperacillin/tazobactam rather than the method stated in the summary of product characteristics (SPC). Practice also varied between critical care networks/regions, with some adopting predominantly EII usage and others using either bolus or short infusion. The only factor associated with EII administration of meropenem was greater pharmacist cover (*p *< 0.001). Adoption of local policy for vancomycin administration by CI was associated with the presence of the pharmacist on the consultant-led ward round (*p *= 0.03).

In 7.8% (5/64) CCUs, EII or CI administration methods were also chosen for patients on renal replacement therapy and in 15.6% (10/64) the administration method was influenced by other patient factors including fluid restriction (2/64), endocarditis (1/64), septic shock (4/64), major burns (1/64) and identification of *Pseudomonas* spp. (2/64). The most commonly stated reason for using alternative administration methods in 48% (31/64) CCUs for using EII/CI was “Evidence Based—pk/pd properties”, with “Evidence based—improved outcomes” also registering highly in 40.6% (26/64) responses. “Cost” was stated in only 3.7% (2/64).

9.4% (6/64) of responders believed the total daily dose of vancomycin differed when using EII/CI: on 2 CCUs they thought they would use a lower overall dose whereas another 4 responders thought they would use a bigger dose. No CCU used EII with the specific aim of reducing the total daily dose of antibacterial required.

## Discussion

We found that there was a great deal of variability in administration practices across responding sites in the UK, although some patterns emerged leading us to identify usual administration practice (greater than 50% of responses) for 17 out of the 22 intravenous antibiotics surveyed, which all followed the method stated in the SPC. The findings from our UK survey square with those reported from the worldwide ADMIN-ICU study [8], though we sought information specifically from critical-care pharmacists over a larger group of antibacterial agents.

Definite trends in choice of antibiotic were found to exist within some classes of antibacterial agents. Of the broad-spectrum penicillins, amoxicillin alone and in combination with clavulanic acid (co-amoxiclav) were used by most units whereas ampicillin was rarely used. Piperacillin/tazobactam was the clear favourite out of the anti-pseudomonal penicillins and meropenem was the most commonly prescribed carbapenem.

There is often a logical theoretical rationale for extending the length of time over which some antibiotics are administered in light of pk/pd studies, but there is currently little published in the way of supportive clinical evidence for this which demonstrates any clear improvement in the risk of further patient morbidity or mortality. We identified three antibacterials which were administered by EII or CI as the usual method of administration (piperacillin/tazobactam, meropenem and vancomycin), which are often prescribed to treat severe infection and/or sepsis.

### Comparison of CI or EII methods over standard administration

In light of the lack of definitive evidence to show that CIs offer an improvement on licensed methods of administration it is interesting to note that approximately half of responding CCUs (49.2%) have adopted CIs as their standard practice. Our findings point to pharmacist input having the biggest influence on the choice of alternative methods of administration—particularly of vancomycin, piperacillin/tazobactam and meropenem. On the other hand, patient orientated factors affected choice of administration in only a small number of responses, such as renal replacement therapy or endocarditis. Therapeutic drug monitoring (TDM) was used to guide therapy in all CCUs using vancomycin regardless of method of administration in line with standard UK practice [9]. It is possible that CIs are favourable for TDM practice allowing greater ease of monitoring.

A prospective multicentre RCT which compared efficacy, safety and cost effectiveness of vancomycin CIs compared with standard therapy [10], showed a shorter time to target concentrations in the CI arm but microbiological or clinical superiority of CIs was not demonstrated. However, CIs showed a 23% cost saving for a 10 day course compared to standard dosing, revealing CI to be a potentially favourable administration option when scaled up. Another study [11] showed that the incidence of nephrotoxicity increases with vancomycin trough concentrations in patients receiving standard dosing, with around 5% incidence when initial trough concentrations is < 10 mg/L and as high as 33% if trough concentration is > 20 mg/L. It is unknown if this effect is caused directly by high trough concentrations, or the associated higher peak or total exposure over the dosing interval, but it is nonetheless of clinical interest as we found that many CI protocols which aim to eliminate peaks and troughs target vancomycin concentrations of 15–25 mg/L. However, the best available evidence to date indicates there is no difference in the incidence of vancomycin nephrotoxicity between standard and CI methods of administration [12].

Meropenem and piperacillin/tazobactam are generally administered every 8 h and have half-lives of approximately 1 h in patients with normal renal function, leaving extended between-dose periods of time below minimum inhibitory concentration (MIC). This is considered to be a contributing factor in treatment failure and the development of resistance to these agents [13]. Bigger and/or more frequent doses or the use of EIIs or CIs have been suggested as ways of improving clinical outcome and reducing resistance [14, 15].

Meropenem and piperacillin/tazobactam are both currently licensed in the UK to be administered as short infusions with a suggested duration of 30 min, and meropenem is also licensed for administration as a bolus injection over 5 min. Advice to give bolus piperacillin/tazobactam was removed from the UK SPC in 2011 to harmonise with the rest of the EU where it is licensed only for short infusion [16]. The pk/pd benefits of CIs and EIIs to these agents has been reviewed [17, 18] and recent RCTs have shown there may be a patient benefit in these alternative administration methods [6, 19] and although the BLING II study [7] did not show any benefit to patient outcomes from continuous infusions, the findings of a 2018 meta-analysis [20] support the use of continuous infusions of piperacillin-tazobactam in critically ill patients to reduce mortality and improve the rate of clinical cure.

### Factors influencing use of alternative dosing methods

Where EIIs or CIs were not the “usual” method of administration in a given CCU, some respondents stated that under certain circumstances they would recommend or use these methods. The reasons stated could be split into two categories. The first would be classed as patients with conditions that are perceived to have significantly altered the pk/pd parameters such as septic shock and major burn injury. Both of these conditions cause an increase in volume of distribution and an increase in renal clearance of water soluble, low protein bound drugs such as the β lactams being discussed [21]. Multiple studies have shown drug plasma levels to be altered in these patient groups [22–24] and others have shown that EIIs or CIs lead to a more favourable pk/pd profile [25]. It is therefore logical that prescribers may target the patients who are likely to gain the most from these practices. The second area is in targeting organisms that have the highest MICs and therefore require high concentrations of antimicrobial at the target site: 2 CCUs used EIIs of piperacillin/tazobactam and meropenem solely for the treatment of *Pseudomonas spp.* which have a high MIC and are noted for high treatment failure rates and development of resistance [26]. Many studies that have investigated EIIs of β-lactams used their ability to maintain levels 4-times greater than the MIC of *Pseudomonas spp.* as the target measure [27, 28]. Again, this supports treatment of *Pseudomonas spp.* with EIIs.

### Factors influencing choice of administration method

Almost half of respondents stated that a major influencing factor was the evidence-base supporting the pk/pd benefits of EIIs and CIs, but a large proportion of the same respondents also stated that they believed the published evidence demonstrated improved patient outcomes. We found a lack of evidence to corroborate this in the literature in contrast to the respondents’ opinions, which may indicate that some pharmacists have only a superficial grasp of the evidence. 13 respondents stated reduced vancomycin toxicity as a driving force for choice. Respondents reported that CIs of vancomycin are generally more convenient—easier for the nursing staff to manage, simpler dose adjustments, timing of TDM less important so interpretation of the plasma level is also easier and therefore under/over- dosing easier to prevent. Interestingly, only 2 respondents stated cost saving as a driving force. Cost improvement plans and means of saving on drug budgets are necessarily towards the forefront of most pharmacists’ minds during every working day in practice and given that many published studies, particularly from the USA, report that lower total daily doses are required when EIIs or CIs are used, it is perhaps surprising more respondents didn’t rate this as a more important consideration. For example, Grant et al. [29] investigated using a loading dose of 4.5 g piperacillin/tazobactam followed by a CI of 9 g per day rather than the licensed dose of 4.5 g three or four times daily, finding similar clinical efficacy but a treatment course cost of $399.38 for CIs vs. $523.49 for licensed practices where shorter infusion times are employed.

### The role of therapeutic drug monitoring

TDM is universally carried out for aminoglycosides (e.g. gentamicin) and glycopeptides (e.g. vancomycin). In the UK, TDM is a key part of standard care for these antibiotics because of their nephrotoxic and ototoxic nature and narrow therapeutic drug index, and more recently with vancomycin to ensure serum trough levels are maintained between 15 and 20 mg/L, or that 24-h AUC:MIC ratio > 400. Given the ease of calculation of AUC for continuous infusions of vancomycin, CI offers a real advantage in improving patient outcomes. Many centres outside the UK do not incorporate any TDM into antibiotic therapy as a matter of course, with 20% of respondents stating they did not monitor gentamicin serum concentration at all, and a further 19% only did so if the patient was in renal failure [8]. The DALI study [30] demonstrated that TDM is especially important in personalising β-lactam antibiotic dosing and administration in critically ill patients, to prevent adverse patient outcomes arising from inadequate dosing.

### Limitations of the study

The response rate for the survey is relatively low despite attempts to follow up potential participants, and responses are largely confined to English CCUs. Although this limits the impact of the findings, the responses do provide a useful picture of the administration practice of antibiotics in UK CCUs, especially in revealing where there is lack of consistency or “standard” across UK NHS Trusts.

## Conclusion

‘Usual’ intravenous administration practices exist for most antibiotics used in UK CCUs, with deviation from the SPC mainly limited to three antibacterial agents with pk/pd profiles that particularly lend themselves to extended infusions and to minimise perceived risk. CCUs tended to follow licensed/SPC methods of administration and where there was no usual practice this was because there was a split in responses across multiple licensed methods of administration. Further research is needed to determine whether extended infusion methods offer clinically meaningful advantages over shorter licenced administration methods in CCU patients.
